# Transcriptome Reveals Granulosa Cells Coping through Redox, Inflammatory and Metabolic Mechanisms under Acute Heat Stress

**DOI:** 10.3390/cells11091443

**Published:** 2022-04-25

**Authors:** Abdul Sammad, Hanpeng Luo, Lirong Hu, Huabin Zhu, Yachun Wang

**Affiliations:** 1 National Engineering Laboratory for Animal Breeding, Key Laboratory of Animal Genetics, Breeding and Reproduction, MARA, College of Animal Sciences and Technology, China Agricultural University, Beijing 100193, China; drabdulsammad1742@yahoo.com (A.S.); luohanpeng@cau.edu.cn (H.L.); b20193040324@cau.edu.cn (L.H.); 2 Embryo Biotechnology and Reproduction Laboratory, Institute of Animal Sciences, Chinese Academy of Agricultural Sciences, Beijing 100193, China

**Keywords:** granulosa cells, heat stress, apoptosis, oxidative stress, steroidogenesis, NF-κB pathway, p53 pathway, AMPK pathway

## Abstract

Heat stress affects granulosa cells (GCs) and the ovarian follicular microenvironment, causing poor oocyte developmental competence and fertility. This study aimed to investigate the physical responses and global transcriptomic changes in bovine GCs to acute heat stress (43 °C for 2 h) in vitro. Heat-stressed GCs exhibited transient proliferation senescence and resumed proliferation at 48 h post-stress, while post-stress immediate culture-media change had a relatively positive effect on proliferation resumption. Increased accumulation of reactive oxygen species and apoptosis was observed in the heat-stress group. In spite of the upregulation of inflammatory (*CYCS*, *TLR2*, *TLR4*, *IL6*, etc.), pro-apoptotic (*BAD*, *BAX*, *TNFSF9*, *MAP3K7*, *TNFRSF6B*, *FADD*, *TRADD*, *RIPK3,* etc.) and caspase executioner genes (*CASP3*, *CASP8*, *CASP9*), antioxidants and anti-apoptotic genes (*HMOX1*, *NOS2*, *CAT*, *SOD*, *BCL2L1*, *GPX4,* etc.) were also upregulated in heat-stressed GCs. Progesterone and estrogen hormones, along with steroidogenic gene expression, declined significantly, in spite of the upregulation of genes involved in cholesterol synthesis. Out of 12,385 differentially expressed genes (DEGs), 330 significant DEGs (75 upregulated, 225 downregulated) were subjected to KEGG functional pathway annotation, gene ontology enrichment, STRING network analyses and manual querying of DEGs for meaningful molecular mechanisms. High inflammatory response was found to be responsible for oxidative-stress-mediated apoptosis of GCs and nodes towards the involvement of the NF-κB pathway and repression of the Nrf2 pathway. Downregulation of *MDM4*, *TP53*, *PIDD1*, *PARP3*, *MAPK14* and *MYC,* and upregulation of *STK26*, *STK33*, *TGFB2*, *CDKN1A* and *CDKN2A,* at the interface of the MAPK and p53 signaling pathway, can be attributed to transient cellular senescence and apoptosis in GCs. The background working of the AMPK pathway through upregulation of *AKT1*, *AMPK,* *SIRT1*, *PYGM*, *SLC2A4* and *SERBP1* genes, and downregulation of *PPARGCIA*, *IGF2,* *PPARA*, *SLC27A3*, *SLC16A3*, *TSC1/2*, *KCNJ2*, *KCNJ16,* etc., evidence the repression of cellular transcriptional activity and energetic homeostasis modifications in response to heat stress. This study presents detailed responses of acute-heat-stressed GCs at physical, transcriptional and pathway levels and presents interesting insights into future studies regarding GC adaptation and their interaction with oocytes and the reproductive system at the ovarian level.

## 1. Introduction

Events of heat waves are common in tropical, subtropical and even in temperate zones due to climate-change-associated global warming [1,2,3]. Heat stress poses challenges to agricultural production and thus the food security [4,5,6], general health and well-being [7,8] and sustainable dairy production [9,10,11]. Ambient temperatures exceeding 25 °C cause heat stress in mammals including cattle [12,13], whereas high temperature in the range 35–40 °C causes enormous alterations in the physiology and biochemistry of the body [13,14,15,16]. Heat-stress-associated biochemical changes in the reproduction system of cattle leads to delayed conception and low fertility rates [17,18,19].

Granulosa cells are the integral component of the ovaries, where they tend to nourish the developing oocyte inside the follicle and regulate the endocrine signals of the reproduction at the ovarian axis [20,21]. Heat stress is shown to markedly decrease the survival rate of bovine granulosa cells (GCs), increase oxidative stress, limit cell proliferation and transition processes, inhibit steroidogenesis and promote GC apoptosis [22,23,24]. Impairment of GCs through various external or internal stress stimuli may compromise the ovarian functions and development competence of oocytes [25,26]. In a previous study, higher ambient temperatures and associated heat stress were shown to be associated with a decline in ovarian reserves and symptoms of reproductive aging in women [27]. In particular, the pre-ovulation stage of the ovarian follicle remains highly susceptible to the adverse effects of heat stress causing oxidative stress and damage to the developing oocytes [28,29,30]. Heat stress causes energetic metabolism alterations in the body [31] and resulting high non-esterified fatty acids (NEFAs), ketones and inflammatory cytokines alter the biochemical profile of the ovarian follicles [18,30,32,33,34]. These biochemical and inflammatory changes cause dysfunction of GCs and lead to developmental competence of the oocytes [30,35,36].

Transcriptomic-level studies involving global RNA-sequencing may give essential biological insights into the cellular mechanisms, metabolic level changes and apoptotic and antioxidant pathways [23]. Earlier studies have reported a senescence in proliferation and reported variable ability of camelid GCs to recover their proliferation potential following acute and chronic heat stress exposure [35,36]. In our previous study about GCs [23], there was a disparity among different temperature ranges where 41 °C presented less lethality than 40 °C. Therefore, it was imperative to evaluate the physiological and transcriptome-level changes in acute heat-stressed GCs. This study will help further our understanding of basic molecular phenomena underlying heat-stress responses in the mammalian body and help us to explore intervention avenues for welfare and general and reproductive health management. Henceforth, in this study we selected 43 °C at the rate of 2 h [37] to inflict acute heat stress to GCs. To the best of our knowledge, there is a lack of systematic transcriptomic research regarding granulosa cell culture in response to acute heat stress (43 °C). We hypothesized that GCs under acute heat stress would exhibit physical insults and undergo extensive changes in biological pathways funneled towards the cellular homeostasis mechanisms.

## 2. Methods

### 2.1. Granulosa Cell Culture, Heat Treatment and Cell Proliferation Assay

Cell collection, culture and treatment methods followed our previous study [38]. In brief, follicular contents were collected from small follicles (3–8 mm) of healthy cyclic bovine ovaries through an 18-gauge syringe and sieved through a 40 µm filter (Corning Inc., Corning, NY, USA). Follicular fluid was centrifuged, supernatant discarded, and GC pellets were washed twice. GCs were seeded on sterile culture plates (Corning Inc., Corning, NY, USA). Culture media consisting of DMEM/F12 medium containing 10% FBS (both from Thermo Fisher Scientific, Waltham, MA, USA) and 1% antibiotic were cultured under 38 °C and 5% CO_2_ in an incubator. GCs were cultured for 48 h and afterwards subjected to 2 h of acute heat stress (43 °C), while the control group remained at 38 °C.

Cells (2 × 10^4^ in each well) were cultured in 96-well plates and optical density (OD) was measured using a Cell Counting Kit-8 (CCK-8; Dojindo Laboratories, Kumamoto, Japan) according to the manufacturer’s instructions. In brief, 10 µL of CCK-8 was added to each well and incubated for 2 h. Post-treatment absorbance of cells was measured at a 450 nm wavelength by a plate reader, followed by measurements at the intervals of 6, 12, 24, 48, 72, 96 and 144 h. For the immediate CCK-8 assay (0 h after heat stress), CCK-8 solution was added just before the start of heat stress treatment. 

### 2.2. Reactive Oxygen Species, Apoptosis and Hormone Measurements

Reactive oxygen species, apoptosis and hormone measurement methods followed our previous study [38]. Cells were cultured in 96-well black plates and fluorescence OD was measured using a 6-carboxy-2′,7′-dichloro-dihydro-fluorescein diacetate kit (DCFDA kit, Abcam, Cambridge, MA, USA) according to the manufacturer’s instructions. Briefly, cells (1 × 10^4^ in each well) were initially cultured, the medium was removed and wells were washed once with 100 µL/well of 1 × buffer (supplied in kit) followed by the addition of 100 µL/well of the 20 μM DCFDA solution. Cells were incubated for 40 min at 38 °C in the dark. After that, DCFDA solution was removed and cells were supplemented with 100 μL/well complete culture medium and incubated for 8 h (heat stress group, 2 h at 43 °C followed by 6 h at 38 °C). After incubation, fluorescence OD was immediately measured on an Infinite M200 PRO (Tecan Deutschland GmbH, Crailsheim, BW, Germany) fluorescence plate reader at excitation/emission = 485/535 nm wavelengths. 

The qualitative apoptosis rate of GCs was measured by a fluorescence microscope with an Annexin V-FITC kit (Nanjing Jiancheng Bio Inst., Nanjing, China). After post-heat-stress recovery at 38 °C, cells were harvested by 0.25% trypsin and washed twice with warm PBS and then added to 500 μL 1 × binding buffer. Subsequently, 5 μL FITC and PI were added to each replicate. After 20 min incubation at room temperature, slides were prepared and observed under a fluorescence microscope (Axio Imager A2; ZEISS Microscopy, Oberkochen, BW, Germany). Fluorescence microscope pictures at 40× of early apoptotic (green) and late apoptotic (red) cells were recorded. Four replicate slides of each were counted for distinct red and green features.

After 6 h of post-heat-stress recovery at 38 °C, the culture media from both groups were collected and immediately stored at −80 °C for the determination of P4 and E2 concentration through a commercial ELISA kit (Cusabio Technology LLC, Wuhan, Hubei, China) according to manufacturer’s protocols. The absorbance was measured by an Infinite M200 PRO (Tecan Deutschland GmbH, Crailsheim, BW, Germany) plate reader at a 450 nm wavelength.

### 2.3. Statistical Analysis of Physiological Parameters

The data from at least six replicates each for cell proliferation, ROS and apoptosis measurements were analyzed using Graphpad Prism version 9.0.0 for windows (GraphPad Software, Inc., San Diego, CA, USA). Analysis of variance was carried out and means were compared through Tukey’s honestly significant difference (HSD) test at a 5% level of significance (α = 0.05). All the data presented in the figures are expressed as mean ± standard error (S.E).

### 2.4. RNA Sequencing

The RNA was isolated from GCs according to the manufacturer’s instructions of TRIzol Reagent method [39]. RNA quality and concentration were assessed using an Equalbit RNA BR Assay Kit (Invitrogen, Carlsbad, CA, USA) and Nanodrop 2000 (Thermo, Waltham, MA, USA). RNA integrity was detected by 1% agarose gel electrophoresis, and it could be used for library construction with 28S/18S > 1. For the RNA-seq library, a total of 2 μg RNA was used for purification and fragmentation using a NEBNext Poly(A) mRNA Magnetic Isolation Module (Cat No. E7490S, New England Biolabs (UK) Ltd., Hitchin, Herts, UK), followed by the cDNA library with the NEBNext Ultra RNA Library Prep Kit for Illumina (Cat No. E7530S, New England Biolabs (UK) Ltd., Hitchin, Herts, UK). Libraries were quantified by Equalbit RNA BR Assay Kit (Invitrogen, CA, USA) and pooled equimolarly, and finally submitted for sequencing by the NovaSeq 6000 System (Illumina, Inc., San Diego, CA, USA), which generated 150 base paired-end reads.

### 2.5. Differential Expression, Validation and Functional Analyses of Differentially Expressed Genes

The quality of the sequencing reads was evaluated by FastQC software (v0.11.9), and global trimming was carried using Fastp (v0.20.0) [40]. All clean reads were mapped to the bovine genome of version ARS-UCD1.2 using the software package STAR (v2.7.5c), and the Picard query (http://broadinstitute.github.io/picard/, accessed on 20 October 2021) was used to mark duplicates. Related statistical summary analysis of RNA-seq data sets of treated and control cells is given in Table 1. We investigated the counts of gene expression through RNA-SeQC software (v2.3.6) [40]. Principal component analysis and clustering structure were performed using the psych and hcluster R packages. For differential expression gene (DEG) screening, the quantile-adjusted conditional maximum likelihood (qCML) was performed using edgeR in the R package [41] with criteria fold change ≥ 1.5 and 0.05 for the alpha of false discovery rate (FDR; 3 samples in treatment group versus 3 samples in control group). 

For RNA-seq data validation, cDNA libraries were constructed using a PrimeScript^TM^ RT reagent kit with gDNA eraser (Perfect Real Time, Takara). Quantitative real-time PCR (RT-PCR) was carried out according to the manufacturer’s protocol of the Sybr Premix EX Taq Kit (Takara, Kyoto, China) using applied Biosystem^®^ StepOnePlus™ (Applied biosystems, CA, USA). Bovine β-Actin and B2M (beta-2-microglobulin) were used as internal control genes. Primers for nine randomly selected significant and non-significant genes were designed using Primer-BLAST (www.ncbi.nlm.nih.gov/tools/primer-blast/index.cgi?LINK_LOC=BlastHome, accessed on 9 February 2022) and double-checked by Oligo v7 (Molecular Biology Insights, Inc., Cascade, CO, USA) [42]. All the primers were synthesized by TsingKe Biological Co., Ltd. (Beijing, China) and are presented in Supplementary Table S1. RNA expression levels relative to the control genes were calculated as 2-△△Ct according to previous research [43]. To compare the results of the RNA-seq analysis and RT-qPCR, fold change values were log2 transformed. 

The DEGs were subjected to the Kyoto Encyclopedia of Genes and Genomes (KEGG) pathway and enrichment analysis of Gene Ontology (GO). Online DAVID software (https://david.ncifcrf.gov/, accessed on 9 February 2022) was used for functional annotation DEGs of the GO terms including biological process (BP), cellular component (CC) and molecular function (MF) enrichment analysis. STRING version 11.5 (https://cn.string-db.org/, accessed on 8 February 2022) was used for the protein–protein interaction (PPI) network analysis of DEGs. 

## 3. Results

### 3.1. Influence of Heat Stress on Physical Parameters of Bovine Granulosa Cells

**Figure 1:**
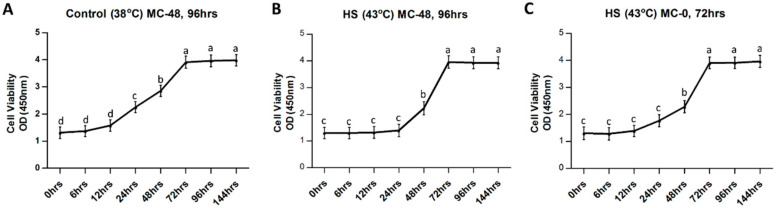
Comparison of physical parameters of bovine granulosa cells (GCs) exposed to heat stress (43 °C for 2 h) versus control (38 °C). GC proliferation curves with mean optical densities (ODs) measured at 450 nm wavelength are plotted against different recovery time points in hours (hrs) for control (**A**), heat stress group with delayed medium change (**B**) and heat stress group with immediate medium change (**C**). Means without common letters (a, b, c, d) are significantly different (*p* < 0.05; *n* = 6 independent cultures). “MC” indicates culture media change at 48 h and 96 h in 1A and 1B, and 0 h (immediately after completion of heat stress in vitro) and 72 h for 1C.

Cultured GCs were exposed to heat stress treatment (43 °C) in vitro, while the control group remained at 38 °C. Cells in the control group maintained steady proliferation activity (Figure 1A). No change in cell viability was observed until 24 h after heat stress exposure (Figure 1B), while the group with medium change at 0 h after heat stress (Figure 1C) did show a certain degree of proliferation activity. Both control and heat stress groups achieved confluences at 72 h after treatment. 

**Figure 2:**
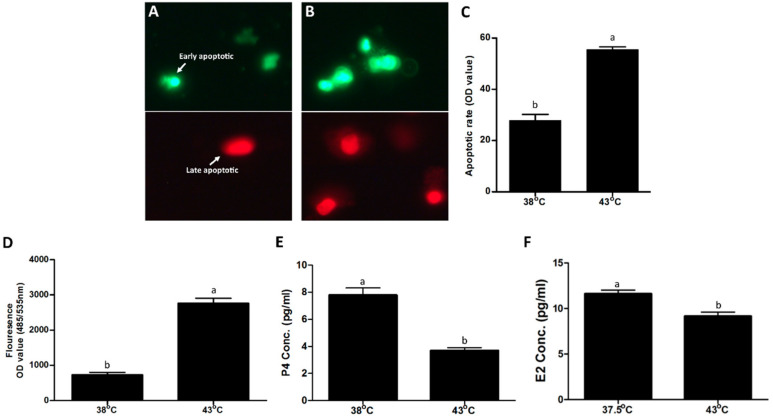
Comparison of physical parameters of bovine granulosa cells (GCs) exposed to heat stress (43 °C for 2 h) versus control (38 °C). Representative fluorescence microscope pictures (200×) of late apoptotic (red) and early apoptotic (green) cells after staining with FITC/PI dye for control (**A**) and heat stress treatment (**B**) groups. Means comparison of apoptotic rate (sum of red and green events) of GCs under control and heat stress (**C**). Reactive oxygen species (ROS) through fluorescence OD value (measured at 485/535 nm wavelength) of GCs stained with 2′,7′-dichlorofluorescine diacetate (DCFDA) is shown on the y-axis, and the temperature treatments are indicated on the x-axis (**D**). Progesterone (P4) and estrogen (E2) concentration change comparison among control and heat stress groups (**E**,**F**), respectively. All data are represented as means ± S.E. of at least three independent cultures with at least three additional replicates for each culture. Means without common letters (a, b) are significantly different (*p* < 0.05).

GCs were subjected to post-treatment exposure recovery for 6 h at 38 °C, after which GC ROS and apoptosis were estimated. Compared to the control group, a significant (*p* < 0.05) increase in ROS level was observed in the heat stress treatment group (Figure 2D). Heat-stressed GCs had a significantly higher (*p* < 0.05) apoptotic rate compared to the control group (Figure 2C), as shown in representative fluorescent microphotographs of early apoptotic and late apoptotic events in the control group (Figure 2A) and heat stress (Figure 2B), respectively. Similarly, P4 and E2 concentration was significantly (*p* < 0.05) decreased in the culture media of the treatment group (Figure 2E,F, respectively).

### 3.2. Role of Acute Heat Stress on GCs through Deep Sequencing

**Figure 3:**
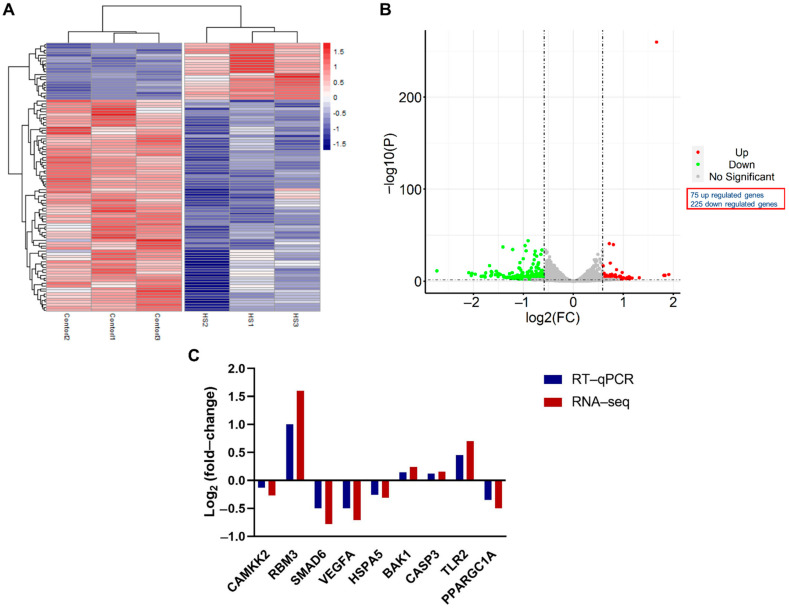
Heatmap for the figure hierarchical cluster analysis of significant differentially expressed genes (DEGs) between heat stress (*n* = 3) and control (*n* = 3) groups of granulosa cells (**A**). Volcano plot of DEGs between heat stress (*n* = 3) and control (*n* = 3) groups (**B**), where red color represents upregulated genes and green color represents downregulated genes (FC ≥ 1.5 and FDR < 0.05). The comparative analysis of the expression level of randomly selected DEGs, using RNA-seq and RT-qPCR (**C**), where DEGs are listed against their log-transformed relative expression fold change values.

RNA-seq read counts of heat stress and control groups (3 replicates each) were distinctly different from each other as determined through principal component analysis and dendrogram clustering structure (Supplementary Figure S1). Whole-genome expression profiling with RNA-sequencing analysis revealed that heat stress had a significant effect on the gene expression in GCs. Out of 12,385 differentially expressed genes (DEGs) listed in Table S2, a total of 330 genes were identified as significant DEGs with a fold change of ≥1.5 and FDR < 0.05 (Table S3). Among these DEGs, 75 were upregulated and 255 were downregulated genes (Figure 3A,B). The log-transformed relative expression fold change of nine randomly selected DEGs in the heat stress and control groups generated from RT-qPCR were in line with the results of RNA-seq data (Figure 3C). The top 20 upregulated and downregulated significant DEGs are listed below in Table 2. The majority of DEGs are involved in cellular stress response, structural remodeling and cellular reprogramming.

**Figure 4:**
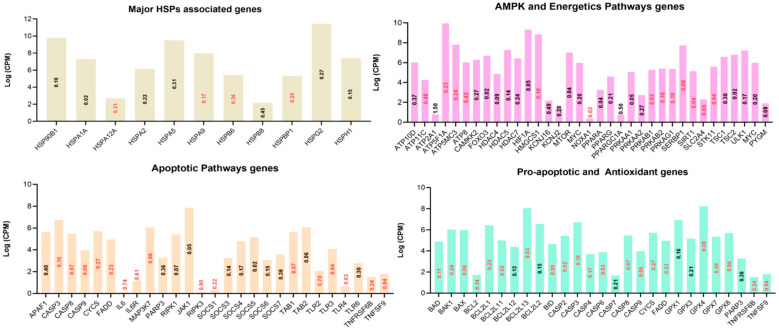
Bar charts based on the manual querying of the differentially expressed genes. Various anti-apoptotic, antioxidant, inflammation, cell damage, chaperone, and cellular-energetic-support-associated genes are given. The y-axis represents the log of counts per million and the log of fold change is presented inside the bars (red upregulated and black downregulated) against respective genes on the x-axis.

**Figure 5:**
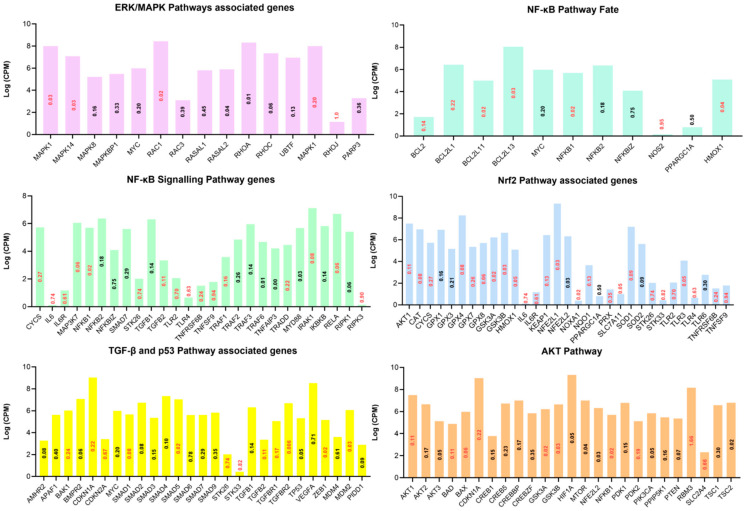
Bar charts based on the manual query of the differentially expressed genes (DEGs). Various important pathways as presented by the titles of the bar charts and associated genes are shown. The y-axis represents the log of counts per million and the log of fold change is presented inside the bars (red upregulated and black downregulated) against respective genes on the x-axis.

Among the several hundred genes up- or downregulated as a result of in vitro acute heat stress (Supplementary Table S2), an effort was made to filter out genes related to the heat shock protein family, apoptosis, antioxidant support, energetic support (Figure 4), and partial clues of major signaling pathways on the basis of extensive manual querying of these pathways in related literature (Figure 5).

### 3.3. Pathway Analysis of Differentially Expressed Genes

**Figure 6:**
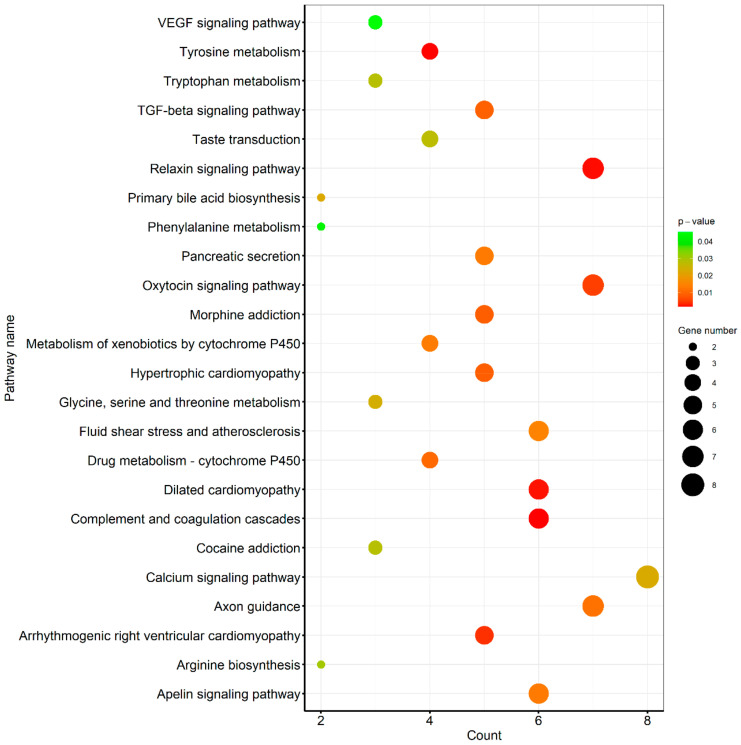
Kyoto encyclopedia of genes and genomes (KEGG)-based pathway analysis of differentially expressed genes (DEGs) in response to heat stress granulosa cells. All the significantly (*p* < 0.05) regulated pathways and DEG enrichment ratios are illustrated.

Detailed results of the KEGG-based pathway analysis of the DEGs are given in Supplementary Table S4. The illustration of all canonical pathways (*p* < 0.05) is given in Figure 6. Additionally, Supplementary Table S4 was checked for the top enriched genes in each pathway, and these are listed along with enrichment ratios (in all pathways) in Table 3.

**Figure 7:**
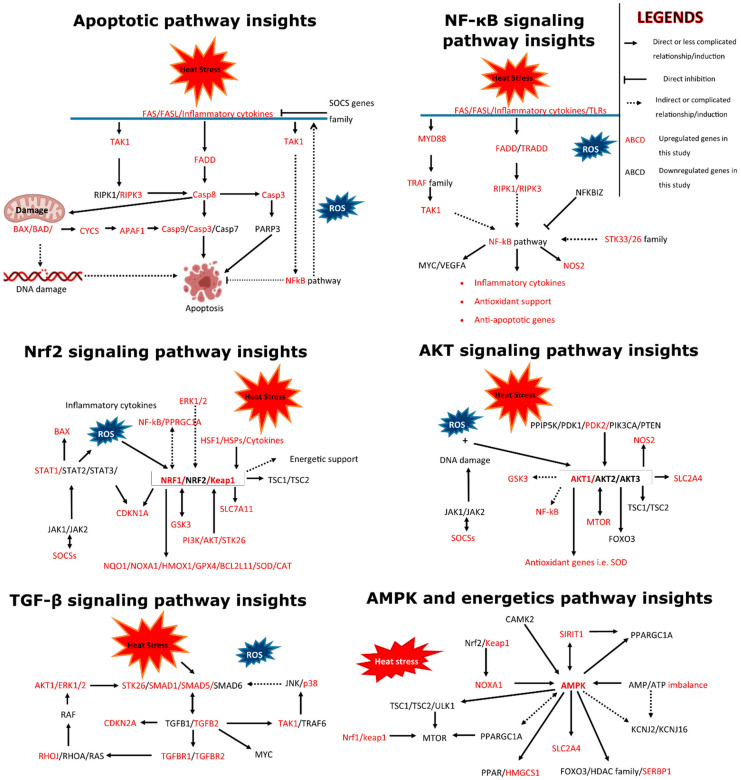
Various important cellular signaling pathways and the genes implicated in these pathways along with the regulation status of these genes in control versus heat-stress treatment of granulosa cells (upregulated genes or gene families are written in red color).

Based on Figure 4 and Figure 5, and a manual search of given pathways in the stress-associated literature and molecular signaling mechanisms given in those studies, Supplementary Table S2 was checked for gene regulation and flowcharts were drawn. Flow charts consisting of various integral cellular pathways and associated genes involved in initiation, intermediate signaling, direct or indirect triggering, target and outcome, and inhibitory mechanisms are given (Figure 7).

### 3.4. Functional Annotation of Differentially Expressed Genes

Further, DEGs were checked for gene ontology (GO) term enrichment. All significant (*p* > 0.05) GO term details given as biological process (BP), cellular component (CC) and molecular function (MF) are presented in Supplementary Table S5. The top enriched genes in each of the functions of BP, CC and MF terms along with their enrichment ratios are listed in Table 4. Genes *TEK*, *VEGFA*, *ACTA2* and *INSL3* have high enrichment ratios in more than one GO term classification, while *ACTA2* is the only upregulated DEG in the given DEGs.

### 3.5. Protein–Protein Interaction (PPI) Networks of DEGs under Acute Heat Stress

**Figure 8:**
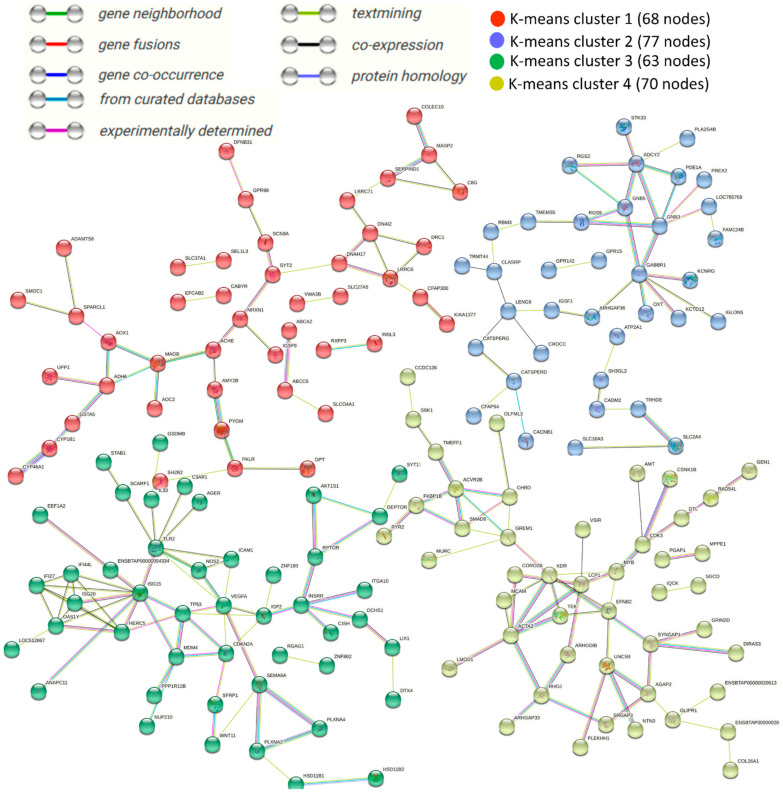
STRING interaction analysis of all differentially expressed genes (DEGs) in control versus heat-stressed granulosa cells. Interaction was performed with confidence score of 0.4. String interaction map is divided into 4 k-means clusters with distinct sets of DEGs. Nodes represent DEGs (protein coding genes) and lines between nodes refer to edges of various sorts of interactions denoted by different colors and defined through the legends present in the figure; standalone nodes lacking edges were removed.

Function protein association network analysis of proteins coding DEGs comprising various k-means clustering groups (STRING) with medium confidence score (0.4) are presented in Figure 8. The total number of nodes was 278, total number of edges (interactions) was 221, average local clustering coefficient was 0.324 and PPI enrichment *p*-value stood at 4.93 × 10^−7^.

Based on the co-occurrence of “from curated database”, “experimentally determined” and “text mining” interaction edges, *SERPIND1*, *MASP2*, *AMY2B*, *PYGM*, *ADH6*, *GSTA5*, *CYP1B1* and *CYP46A1* can be regarded as important nodes in k-means clustering 1 (68 nodes). Similarly, *ADCY2*, *GNB5*, *GNB3*, *RGS9*, *GABBR1* and *SLC2A4* can be regarded as important nodes in k-means clustering 2 (77 nodes). Genes *ISG15*, *TP53*, *MDM4*, *CDKN2A*, *IGF2*, *AK1TS1*, *RPTOR*, *INSRP*, *SEMA6A*, *PLXN2A* and *PLXNA4* can be regarded as important nodes in k-means clustering 3 (63 nodes). *ACVR2B*, *FKBP1B*, *SMAD6*, *KDR*, *ACTA2*, *RHOJ*, *UNC5B* and *SYNGAP1* can be regarded as important nodes in k-means clustering 4 (70 nodes) in a similar way.

**Figure 9:**
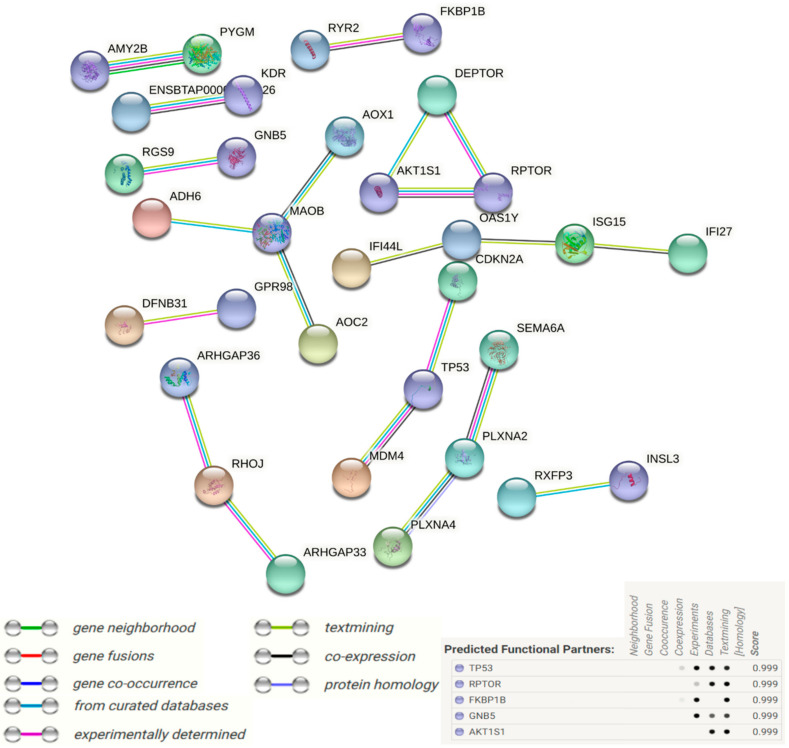
STRING interaction analysis of all differentially expressed genes in control verses heat-stressed granulosa cells. Interaction was performed with a confidence score of 0.9. Nodes represent genes and lines between nodes refer to edges showing various sorts of interactions denoted by different colors and defined through the legends present in the figure; standalone nodes lacking edges were removed. Important nodes with the highest interaction scores of functional predictions are given in the bottom right of the figure.

Additional full STRING network analysis with the highest confidence score (0.9) was also carried out to narrow down the important interaction nodes (Figure 9), while details are given in Supplementary Table S6. *TP53*, *RPTOR*, *FKBP1B*, *GNB5* and *AKT1S1* were the important functional nodes with the highest score. *SERPIND1*, *MASP2*, *AMY2B*, *PYGM*, *MAOB*, *ISG15*, *SEMA6A* and *PLXNA2* can be regarded as important nodes based on their centrality and edges.

## 4. Discussion

Heat stress causes various adverse modifications at physiological and metabolic levels in cows [15,16]. However, the occurrence of atypical negative energy balance in postpartum cows during heat stress is very important, which is characterized by blunt non-esterified fatty acid (NEFA) response [15,31] (keep it like this and remove 47, no need of 47 here, I cannot change these (please do it by yourself), otherwise whole file references and citations will be disturbed) [15,31]. This phenomenon, when accompanied with low body condition scores, clinical ketosis, decreased feed intake, fatty liver and other morbidities, leads to impairment of the reproduction process [44,45,46]. Summer-time heat stress in the presence of negative energy balance and abnormal postpartum conditions [44,45,46] can cause changes in the blood metabolic profile extending effects towards ovarian follicles, developing oocytes and embryos [47,48]. Primary and pre-antral ovarian follicles are particularly susceptible to the adverse effects of heat stress [18,28]; the compromise of GCs in the follicles has a negative impact on oocytes [25,49], leading to a decline in their developmental competence [50]. Hence, we investigated the physical, biochemical and transcriptome-level changes in acute-heat-stressed GCs.

There was a significant decline in cell proliferation potential (cell senescence) in the heat stress group compared to the control group. Previous studies support this finding, where acute-heat-stressed (45 °C) GCs re-attained proliferation potential 48 h post-treatment [35,36,37]. A 24 h delay in cell confluence achievement (at 72 h) observed in our study can be attributed to different experimental methods and the species difference in these previous studies, as camels are thought to be relatively thermo-tolerant [35]. However, the trend in proliferation activity recovery of our GCs follows the results reported by Alemu et al. and Fu et al. [22,37], where they reported post-heat-stress recovery of GC proliferation activity at 48 h. GCs without heat stress treatment proliferated steadily, while the heat stress group with immediate culture media change showed relatively earlier positive proliferation change compared to the heat stress group with culture medium change at 48 h. This phenomenon is interesting and need further investigation; a preliminary hypothesis may be drawn that heat stress is an energy-intensive process, as supported by our previous review of the literature studies [16,51]. Energy balance has a direct relationship with follicular growth and development, ovulation and fertility in postpartum dairy cows [52]. Therefore, careful monitoring of feeding with enough potential to maintain a positive energy balance appears to be the most important intervention avenue in dairy cows exposed to heat stress [15]. NEBAL is the most basic cause for poor reproductive efficiency [13,14,15,16], emphasizing the importance of maintaining positive energy balance manifolds in the case of additional heat stressors [18]. Going back to cell-specific responses to acute heat stress, downregulation of *TP53*, *MDM4*, *MYC*, *PARP3*, *MAPK8* and *RAS2* and upregulation of *MAP3K7, CDKN2A, SMAD6* and *TGFB2* is important. These genes present strong evidence of the involvement of the p53 signaling pathway working in conjunction with other major pathways affecting acute-heat-stressed GC cell proliferation and causing high apoptosis through complex signaling mechanisms [53,54]. Together with other pathways, the p53 signaling pathway can be regarded as one of the major contributing pathways towards the initial high rate of apoptosis and later resumption of cell proliferation at 48 h in heat-stressed GCs [55,56].

Acute heat stress in vitro triggered accumulation of intracellular ROS and caused an increase in apoptosis of GCs in our study; these results are in accordance with the previous studies [23,57]. Previous studies show that post-heat-stress intracellular ROS accumulation increases in a time-dependent manner [22], while our study shows a substantial intracellular ROS accumulation within 10 h of heat stress initiation. ROS production is a normal phenomenon occurring in the cells due to fundamental biological processes [58]. Higher ROS accumulation in the presence of stress stimuli and mitochondrial damage [22] impairs cell antioxidant response elements (AREs) and causes oxidative stress [23,59]. Besides the intracellular high ROS in acute-heat-stressed GCs in our study, other evidence like upregulated sets of *BAX*, *BAK* and *BID*, and *HMOX1, NOXA1, NQO1, BCL2L11, SOD, CAT* and *GPX4*, strongly evidence the presence of oxidative stress and reciprocal cellular mechanisms of a higher antioxidant response against it. Previous studies involving cellular stress associates intercellular ROS with oxidative stress [60] and apoptosis in cells [61,62]. In our study, this high rate of apoptosis was typically characterized by upregulation of *FADD, TRADD, MAP3K7, RIPK3* and caspases. ROS-mediated oxidative stress induces bovine GC apoptosis and compromises fertility in cows [63,64]. However, pathways and mechanisms underlying heat stress causing extreme oxidative stress and blunt ARE response causing apoptotic fates of the cells are not completely understood.

Heat-stressed GCs decreased the production of P4 and E2 hormones, as demonstrated previously [22,23,55]. Lower levels of these hormones in heat stress cause poor estrus behavior through E2 and low luteal support and fertility through P4 levels [18]. This decline in steroidogenic activity of GCs can essentially be attributed to the downregulation of *STAR, CYP11A1* [23] and mitochondrial damage due to ROS [22]. Gene CYP11A1 was also downregulated in our study; however, *STAR* was mildly (Log_2_(FC) = 0.051) upregulated. This phenomenon is interesting as many of its orthologues like STARD13, *STARD8*, *STARD3* and *STARD4* were downregulated in the heat stress group. We further searched other genes related to P4 synthesis and found that *SRD5A1*, *HSD17B11* and *SREBF1* were also downregulated; therefore, we propose a complex transcription mechanism underlying the decreased P4 secretion under heat stress. This claim may be further augmented by our finding about the majority of genes involved in cholesterol synthesis and homeostasis (such as *HMGCS1, HMGCR, ACTA1, ACTA2, ABCA1, SLC25A1, SAOT1, AMPK, SRD5A3, DHDDS NS FDPS*) being upregulated in heat stress. A very similar dilemma was observed with the specific genes involved in E2 synthesis: where *CYP19A1* was upregulated, *CYP17A1* was not detected at all, and *HSD3B1* and *HSD17B1* were downregulated. We could not identify deeper and mechanistic causes underlying these phenomena; however, our results present a further line of investigation. The established phenomenon of blunt NEFA response in heat-stressed cows is characterized by low fat mobilization, as explained by previous studies [15,31,47]; upregulated genes involved in fatty acids and cholesterol metabolism indicate that fatty acid metabolism is an adaptation of heat-stressed GCs. It can be inferred that fat mobilization is disturbed at organism level in heat stress, but cellular metabolism of fats remains enhanced. Therefore, supplemental fats can help to avert the heat-stressed typical NEBAL, as supported by previous studies [65].

As shown earlier in this study, the majority of genes primarily associated with cell damage (*CYCS*), inflammation (*TLR2, TLR4, IL6, TNFRSF6B, TNFSF9*), apoptosis initiation (*BAD, BAX, BAD, BAK1, BID*) and execution (*FADD, TRADD* and caspases) are upregulated in heat stress, while conversely a large proportion of antioxidant and anti-apoptotic genes (*HMOX1, NOXA1, NQO1, SIRIT1, BCL2L11, SOD, CAT, GPX4*) are also upregulated. These variable and complicated phenomena indicate evidence of intricate “biological fight” in heat-stressed GCs, and essentially explain the observation of post-heat-stress transient senescence of GCs found in this and a previous study [35]. 

In the event of cellular stress, the Nrf2 pathway is involved in complex upregulatory functions related to cell metabolism in conjunction with NF-κB (nuclear factor kappa light chain enhancer of activated B cells) [66,67], PI3K/AKT/mTOR [68,69,70] and AMPK signaling pathways [71]. Evidence of high ROS, upregulation of inflammatory cytokines, *ERK1/2*, *AKT1*, *KEAP1* and *NRF1* indicates induction of the Nrf2 pathway, which is augmented by antioxidant and anti-apoptotic genes. Genes *GSK3A*, *GSK3B* and serine threonine kinases like *STK26* and *STK33* also evidence the activity of the Nrf2 pathway. The upregulation of *CDKN2A* is also interesting, and augments the repression of transcription activity by the Nrf2 pathway in heat-stressed GCs [72]. Gene *SLC2A4* encoding GLUT4 was upregulated in heat stress; however, we propose that the Nrf2 pathway cannot be solely implicated in the GC bioenergetics-based protective responses against thermally driven oxidative stress. The complex role of the transforming growth factor-β (TGF-β) pathway is also important in this context, and is involved in signaling mechanisms related to upregulation of NF-κB and inducing apoptosis through cell cycle arrest [73,74]. The most important evidence of cell cycle arrest in heat-stressed GCs can be explained by our finding of the downregulation of *MYC, TP53* and *MDM4* and upregulated *CDKN2A* and *CDKN1A*, which may lead to apoptosis through p53 signaling pathway involvement [53,54]. Similarly, there was evidence of non-canonical induction of the TGF-β signaling pathway as indicated by downregulation of *SMAD6* and upregulation of *RHOJ*, *MAPK1* and *AKT1* in this study [75]. Regarding the MAPK signaling, upregulation of *MAP3K7* (*TAK1*) and *MAPK14* (p38) [54] and downregulation of *MAPK8* are of particular importance [76], as evidenced in this study, and may contribute to the apoptosis of heat-stressed GCs. In the event of cellular stress, the Nrf2 and NF-κB pathways are involved in complex regulatory functions [66,67] related to cell metabolism in conjunction with AMPK signaling pathways [71]. Regarding the AMPK signaling pathway in this study, it is characterized by downregulation of ATP gene families and *PPARGC1A*, and upregulation of *SIRIT1, GLUT4, HMGCS1* and *SERBP1* [71,77].

GCs play an integral role in ovaries through their steroidogenic roles and oocyte development role [20,21]. Heat stress and its adverse effects on GCs have been implicated as one of the causes of fertility decline in mammals [18,23,27]. In the previous section of discussion, we elaborated the effects of acute heat stress on the physical parameters of GCs. At the same time, we tried to elaborate brief insights about the pattern of major biological pathways and their regulation. Out of 12,385 true RNA reads from the global transcriptomic comparison of control and acute-heat-stressed GCs, 330 significant DEGs were determined through post-analyses. The majority of DEGs were downregulated (225), indicating adverse effects of acute heat stress on the transcriptome of GCs. Going through the list of the top 20 up- and downregulated DEGs, *RBM3* appeared as the most significant upregulated DEG; this gene, being a cancer biomarker, is also shown to promote DNA integrity and promote cellular homeostasis through complex signaling [78]. GCs are obviously overstressed and the presence of various cell-structural remodeling (*KASH5, ACTA2, LRRC6, MCAM, PLXNA2, CROCC*), regulation of cell proliferation (*ITGB3BP, MAP3K5, SMAD6, TEK, STK33*), catabolic support (*GPT, GSTA5, PYGM, INSL3, MAOB, METAPID, PKLR, UPP1, AMT, HSD11B1, IGF2, SLC2A4, SLC16A3, SLC27A3*), inflammation (*IL33, ISG20, IGSF9, TP53, TLR2, CDKN2A, MDM4, NFKBID, NOS2, NFKBIZ, TNFSF9*), G-protein coupled receptors (*GPR98, GPR142, GPR15*), GTPase-related genes (*RHOJ, SRGAP3, SYNGAP1, ARHGAP36, RGS9, RGS2*), calcium transport and membrane-channel-related genes (*EFCAB2, SMOC1, CACNB2*), zinc finger proteins and F-box-only protein families of genes explains a major part of the upregulated DEGs. Similarly, the majority of the top 20 downregulated DEGs relates to cell-structural disturbance, lack of cell proliferation potential and genes related to the energetic homeostasis of cells. The heat-shock protein family response in the presence of upregulated *HSF1* was also relatively blunted in response to acute heat stress, with only a few members such as *HSPA12A, HSPA9, HSPB6* and *HSPBP1* being upregulated genes. The details of pro-apoptotic, anti-apoptotic and antioxidant genes have been discussed earlier, where there is evidence of strong ongoing antioxidant response in GCs after acute heat stress. KEGG functional pathway analysis shows an enrichment of pathways, such as VEGF and TGF-β signaling, showing the disturbance of cellular transcription and proliferation. Various amino-acid-metabolism-associated pathway enrichments show the evidence of protein breakdown and the decline in cellular energetics homeostasis. The remaining KEGG-enriched pathway nodes suggest intercellular structure breakdown and disturbance. These observations are augmented by the downregulated status of the entire list of top DEGs enriched in all KEGG pathways. The majority of GO annotation enrichments were observed in the BP enrichment terms, where, except *ACTA2*, all DEGs with the highest enrichment ratios were downregulated in response to acute heat stress. Genes *ACAT2*, *TLR2* and *SLC2A4* are the only three upregulated DEGs in the top 20 different GO term enrichment processes. Earlier studies involving acute heat stress in bovine skeletal muscles [68] and GCs [38] reported altered metabolism and insulin signaling, lack of anabolic activities and extensive reshuffling in the bioenergetics mechanisms involving multiple signaling pathways. Heat stress and associated oxidative stress are shown to lead to upregulated inflammatory response [79,80], while inflammation is energy intensive and leads to metabolic imbalance [81,82]. Similarly, in a previous study involving acute and chronic heat-stressed GCs, there were evident changes in the cell morphology, proliferation potential, and wound healing potential [35], where cells lost their tone and became rounded, and evidence of granules and cytoplasmic fragmentations were visible. Therefore, among these DEGs, *TLR2* can be taken as a representative of inflammatory response and cytokine signaling upregulation, *SLC2A4* as a representative of cellular energetic support mechanisms and *ACTA2* as the representative of the resort of maintaining the cellular structural integrity in response to heat stress. 

## 5. Conclusions

Upon the infliction of in vitro acute heat stress, GCs suffer from decreased cell viability, high oxidative stress and increase in the apoptosis rate. The steroidogenic potential of GCs declines, in spite of the evidence of normal cellular cholesterol metabolism. A large number of antioxidant and anti-apoptotic genes remain upregulated, in spite of strong evidence of upregulated pro-apoptotic and apoptotic genes. There is extensive interplay among all major cell signaling pathways, where the evidence suggests repression of cell transcriptional and proliferation activity, and the cellular “resort” of averting the effects of heat stress through remodeling of cellular structural proteins and energetic homeostasis. The evident role of the inflammation-metabolism nexus appears to be central in the acute-heat-stressed mediated GCs’ ultimate fate, and hence this study indirectly emphasizes this intervention nexus for reproductive management under heat stress. Our RNA-seq approach gives novel insights into the biological mechanisms and transcriptional activity of GCs exposed to acute heat stress and opens gates of further research in these type of cell-stress stimuli interactions. 

## Figures and Tables

**Table 1:** Statistical summary analysis of RNA-seq data sets of treated and control cells.

Samples	Mean Reads			Ratios (%) for Mapping		
	Raw Reads	Valid Reads	Q20 (%)	Uniquely Mapped	Mapped to Multiple Loci	Mapped Reads
Control	43,955,930	43,547,344	97.99%	91.92%	1.27%	93.19%
Control	44,851,996	44,429,040	98.04%	87.19%	1.28%	88.47%
Control	44,353,984	43,930,404	97.86%	93.37%	1.27%	94.64%
Treated	51,330,838	50,826,320	97.87%	93.58%	1.29%	94.87%
Treated	41,539,088	41,174,768	97.95%	93.25%	1.28%	94.53%
Treated	50,037,758	49,578,770	98.03%	92.40%	1.26%	93.66%

**Table 2:** Detailed list of top 20 upregulated and downregulated significant differentially expressed genes (DEGs) between heat stress (*n* = 3) and control (*n* = 3) groups of granulosa cells (GCs).

DEGs	LogFC	LogCPM	FDR	Description
*RBM3*	1.66	8.16	2 × 10^−256^	RNA binding motif protein 3
*GREM1*	0.72	6.91	5 × 10^−38^	“gremlin 1, DAN family BMP antagonist”
*ACTA2*	0.80	7.10	5 × 10^−37^	“actin alpha 2, smooth muscle”
*IFI27*	0.74	5.16	6 × 10^−18^	putative ISG12(a) protein
*LCP1*	0.60	5.67	4 × 10^−15^	lymphocyte cytosolic protein 1
*ARHGDIB*	0.86	3.50	5 × 10^−11^	Rho GDP dissociation inhibitor beta
*IQCK*	0.98	2.54	4 × 10^−8^	IQ motif containing K
*PCSK1*	0.62	3.98	2 × 10^−7^	proprotein convertase subtilisin/kexin type 1
*DTL*	0.61	3.74	8 × 10^−7^	denticleless E3 ubiquitin protein ligase homolog
*ADH6*	0.77	3.04	1 × 10^−6^	alcohol dehydrogenase 6 (class V)
*PYGM*	1.91	0.08	3 × 10^−6^	“glycogen phosphorylase, muscle associated”
*C3AR1*	0.75	2.90	4 × 10^−6^	complement C3a receptor 1
*PREX2*	0.84	3.25	5 × 10^−6^	phosphatidylinositol-3,4,5-trisphosphate dependent Rac-exchange factor 2
*HERC5*	0.73	3.17	6 × 10^−6^	HECT and RLD domain containing E3 ubiquitin protein ligase 5
*SPARCL1*	0.66	3.38	7 × 10^−6^	SPARC like 1
*AOX1*	0.64	3.64	1 × 10^−5^	aldehyde oxidase 1
*ADAMTS20*	1.81	0.24	2 × 10^−5^	ADAM metallopeptidase with thrombospondin type 1 motif 20
*KASH5*	1.84	0.33	2 × 10^−5^	KASH Domain Containing 5
*LOXL4*	0.80	2.89	2 × 10^−5^	lysyl oxidase like 4
*CDKN2A*	0.67	3.42	3 × 10^−5^	cyclin dependent kinase inhibitor 2A
*LENG8*	−0.91	7.45	6 × 10^−41^	leukocyte receptor cluster member 8
*DCHS1*	−0.97	5.88	5 × 10^−36^	dachsous cadherin-related 1
*CCDC136*	−1.21	4.19	6 × 10^−32^	coiled-coil domain containing 136
*PLXNA2*	−0.63	6.69	2 × 10^−31^	plexin A2
*MPPE1*	−0.95	5.00	1 × 10^−30^	metallophosphoesterase 1
*TBX18*	−0.65	6.37	5 × 10^−27^	T-box transcription factor 18
*SMAD6*	−0.78	5.61	1 × 10^−26^	SMAD family member 6
*ABCC5*	−0.74	5.41	4 × 10^−25^	ATP binding cassette subfamily C member 5
*SCN9A*	−1.00	4.03	2 × 10^−22^	sodium voltage-gated channel alpha subunit 9
*RSRP1*	−1.07	4.17	2 × 10^−18^	arginine and serine rich protein 1
*CROCC*	−0.70	5.30	3 × 10^−18^	“ciliary rootlet coiled-coil, rootletin”
*EFNB2*	−0.81	4.55	5 × 10^−17^	ephrin B2
*VEGFA*	−0.71	8.52	3 × 10^−16^	vascular endothelial growth factor A
*DTX4*	−1.68	1.96	3 × 10^−15^	deltex E3 ubiquitin ligase 4
*KLF7*	−0.74	4.37	2 × 10^−13^	Kruppel like factor 7
*SEL1L3*	−0.73	4.70	2 × 10^−13^	SEL1L family member 3
*PPP1R12B*	−0.84	3.81	7 × 10^−13^	protein phosphatase 1 regulatory subunit 12B
*RGS9*	−1.09	3.03	9 × 10^−13^	regulator of G protein signaling 9
*KIFC2*	−0.69	4.63	3 × 10^−12^	kinesin family member C2
*GABBR1*	−0.63	4.31	2 × 10^−10^	gamma-aminobutyric acid type B receptor subunit 1

FDR; false discovery ratio.

**Table 3:** Pathway analyses of differentially expressed genes in response to heat stress, checked for top 10 DEGs abundantly enriched in various pathways, are listed along with their statistics details and descriptions.

Genes	E.R.	LogFC	LogCPM	*p*-Value	Description
*ADCY2*	7	−0.73	2.83	2 × 10^−5^	adenylate cyclase 2
*RYR2*	7	−0.88	1.33	3 × 10^−5^	ryanodine receptor 2
*MAOB*	6	−0.82	1.47	4 × 10^−3^	monoamine oxidase B
*ATP2A1*	5	−1.50	0.80	3 × 10^−7^	ATPase sarcoplasmic Ca^2+^ transporting 1
*VEGFA*	4	−0.71	8.52	3 × 10^−16^	vascular endothelial growth factor A
*CACNB1*	4	−0.81	3.07	8 × 10^−7^	Ca^2+^ voltage-gated channel subunit beta 1
*GNB3*	4	−0.91	1.09	5 × 10^−3^	G protein subunit beta 3
*NOS2*	4	0.95	0.16	2 × 10^−2^	nitric oxide synthase 2
*AOC2*	3	−0.91	1.68	2 × 10^−4^	amine oxidase copper containing 2
*KDR*	3	−1.05	0.02	1 × 10^−2^	kinase insert domain receptor

E.R., enrichment ratio in separate KEGG pathways.

**Table 4:** Detailed annotation of gene ontology terms (GO) of significant differentially expressed genes (DEGs) in control versus acute heat stress treatment groups was carried out. Top 10 DEGs enriched in various terms of biological process (BP) and top 5 enriched DEGs in cellular component (CC) and molecular function (MF) are detailed along with their term enrichment ratio and statistical detailing.

Top 10 Genes in Biological Process (BP) of Gene Ontology Enrichment Terms					
Genes	TER	LogFC	LogCPM	FDR	Description
*SFRP1*	61	−0.66	1.83	0.011	secreted frizzled related protein 1
*TEK*	54	−0.97	1.22	0.001	TEK receptor tyrosine kinase
*VEGFA*	49	−0.71	8.52	3 × 10^−16^	vascular endothelial growth factor A
*DRC1*	13	0.94	0.17	0.016	dynein regulatory complex subunit 1
*LRRC6*	15	−0.70	1.39	0.016	leucine rich repeat containing 6
*LFNG*	15	−0.62	4.30	8 × 10^−7^	LFNG O-fucosylpeptide 3-beta-N-acetylglucosaminyltransferase
*INSL3*	10	−1.40	0.40	3 × 10^−5^	Insulin Like 3
*CATSPERD*	10	−0.83	3.17	7 × 10^−9^	cation channel sperm associated auxiliary subunit delta
*NFKBIZ*	10	−0.75	4.09	1 × 10^−9^	NFKB inhibitor zeta
*ACTA2*	9	0.80	7.10	5 × 10^−37^	“actin alpha 2, smooth muscle”
**Top 5 genes in cellular component (CC) of gene ontology enrichment terms**					
*TEK*	7	−0.97	1.22	0.001	TEK receptor tyrosine kinase
*ACTA2*	5	0.80	7.10	5 × 10^−37^	“actin alpha 2, smooth muscle”
*SLC2A4*	3	0.66	2.31	0.004	solute carrier family 2 member 4
*CAVIN4*	3	−0.67	1.21	0.023	caveolae associated protein 4
*TLR2*	2	0.70	2.06	0.005	toll like receptor 2
**Top 5 genes in molecular function (MF) of gene ontology enrichment terms**					
*IGF2*	7	−0.98	0.20	0.032	insulin like growth factor 2
*OXT*	6	−1.51	0.13	0.0002	oxytocin
*APLN*	6	−1.01	0.49	0.003	apelin
*INSL3*	6	−1.40	0.40	3 × 10^−5^	Insulin Like 3
*VEGFA*	5	−0.71	8.52	3 × 10^−16^	vascular endothelial growth factor A

TER; term enrichment ratio (frequency of occurrence in various components) of genes, FDR; false discovery ratio.

## Data Availability

All the pertinent data including those of RNA-seq are presented in the manuscript and associated Supplementary Files.

## Supplementary Materials

The following are available online at https://www.mdpi.com/article/10.3390/cells11091443/s1, Figure S1: RNA-seq read counts of heat stress and control groups (3 replicates each) are distinctly different from each other as shown in principal component analysis and dendrogram clustering structure. Table S1: List of primers used for RT-qPCR validation of genes found in RNA sequencing. Table S2: List of all differentially expressed gene reads along with their statistics parameters, found in control versus heat-stressed granulosa cells. Table S3: List of all significant (*p* < 0.05) differentially expressed genes (DEGs) in response to heat stress in granulosa cells. Table S4: Kyoto encyclopedia of genes and genomes (KEGG)-based pathway analysis of differentially expressed genes (DEGs) in response to heat-stressed granulosa cells. All the significantly (*p* < 0.05) regulated pathways and DEG enrichment ratios are illustrated. Table S5: Detailed annotation of gene ontology (GO) terms (*p* > 0.05) of significant differentially expressed genes in control versus acute heat-stress treatment groups. GO terms including biological process (BP), cellular component (CC), and molecular function (MF) enrichment ratios are given. Table S6: List containing differentially expressed genes and their statistical parameters involved in PPI interaction network analysis at the confidence score of 0.9.
