# Supplementary material for: Transcriptome Reveals Granulosa Cells Coping through Redox, Inflammatory and Metabolic Mechanisms under Acute Heat Stress
Source: Cells. 2022 Apr 25;11(9):1443. doi: 10.3390/cells11091443 (PMC9105522; doi:10.3390/cells11091443)
Supplement: Supplementary file 1 [file cells-11-01443-s001.zip › Supplementary Figure S1 for proof.pdf]

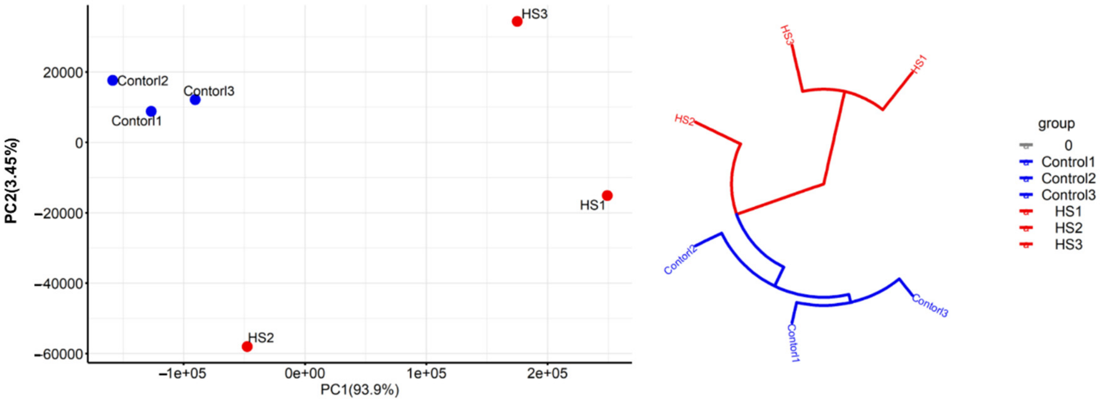

Supplementary Figure S1: RNA-seq read counts of heat stress and control groups (3 replicate each) are distinctly different from each other as shown in principal components analysis and dendrogram clustering structure.
