# Supplementary material for: Transcriptome Reveals Granulosa Cells Coping through Redox, Inflammatory and Metabolic Mechanisms under Acute Heat Stress
Source: Cells. 2022 Apr 25;11(9):1443. doi: 10.3390/cells11091443 (PMC9105522; doi:10.3390/cells11091443)
Supplement: Supplementary file 1 [file cells-11-01443-s001.zip › Supplementary Table S1 for proof.pdf]

Table S1: List of primers used for RT–qPCR validation of genes found in RNA sequencing.

Gene	Accession no.	Forward 5′→3′	Reverse 5′→3′
BAX	NM_173894.1	AGACACCTGAGCTGACCTTG	GTCCCGAAGTAGGAGAGGAG
CASP3	NM_001077840.1	CTGGACTGTGGCATTGAGAC	GCAAAGGGACTGGAGAACC
RBM3	NM_001303463.1	GTCCAGAGACTATGGTGGCAG	TCATTTGGAGAGGTGGTCCTG
CAMKK2	XM_587244	TGGAGACGAGTATTGCGACA	CGCCCAACGTAGTCAAACTT
VEGFA	NM_174216.2	ATGACGAAAGTCTGGAGTGTG	TCTCCTATGTGCTGGCTTTG
HSPA5	NM_001038505.2	CAACCAACTGTTACCATCAAGG	AAAGGTGACTTCAATCTGTGG
BAK1	NM_001077918.1	AGAACCTAGCAGCACCAT	CGATCTTGGTGAAGTACTC
β-actin	NM_173979.3	AACTTGCGCAGAAAACGAGA	CTGTCACCTTCACCGTTCCA
TLR2	NM_174197.2	CATGGGTCTGGGCTGTCATC	TCACACACCTCTGCAGGTCTC
B2M	NM_173893.3	GGGCTGCTGTCGCTGTCT	CTTCTCCCCATTCTTCAGCAAA
PPARGC1A	861 bp length	TCCCCAGGCAGTAGATCTTC	TCCTCGTAGCTGTCATACCTG
